# Dr. Bulwer's Pathomyotomia: A Seventeenth-Century Work on the Expression of the Emotions

**Published:** 1904-09-17

**Authors:** 


### Dr. Bulwer's Pathomyotomia:

### A SEVENTEENTH-CENTURY WORK ON THE EXPRESSION OF THE EMOTIONS.

In a previous article (p. 418) we gave a short account of Dr. Bulwer's "Man Transformed," perhaps the best known of his works because it deals with matters of popular interest. The "Pathomyotomia" is more scientific, and is of much more lasting value. It shows Bulwer to have been a man far in advance of his time, of whose life and training we would gladly know more. The "Pathomyotomia" is a small *duodecimo*, dedicated by the author "to my loving father, Mr. Thomas Bulwer, with a certain filial decency in regard the argument of it is provincial to Physick, wherein your experience hath crowned your Profession, having ever been *fortunatus in praxi*." The sub-title of the work is "A Dissection of the Significative Muscles of the Affections of the Mind, being an Essay to a new method of observing the most important movings of the muscles of the Head as they are the neerest and immediate organs of the voluntarie or impetuous motions of the Mind; With the proposall of a new Nomenclature of the Muscles." Throughout the treatise the author shows himself to be an expert anatomist, skilled in dissection but yet learned in the writings of the classical anatomists, from Galen to Vesalius and Fabricius. The new nomenclature of the muscles is based upon the uses of the muscles, and is therefore physiological in character. Yet it is curious to notice that although his mind has a strong physiological bent, he leans to the old and not to the modern physiology. Writing in 1649, and his father still alive, he could not then have been a man of more than middle age, yet he never mentions Harvey, and when he speaks of the heart he says, "You would verily think that the Heart was a muscle and the grand principle that set all the other muscles in motion: whereas it is known well enough that the heart is neither a muscle nor of itself can move the muscles, for that moves only the arteries," yet Harvey had shown the use of the heart forty years previously. But Bulwer may be forgiven all his errors, because of the care with which he has studied the Anatomy of Expression and of the emotions. He gives a first-rate chapter on Laughter in all its varieties, and if he did not accept the newer teaching, his ideas were even more unacceptable to his contemporaries than were those promulgated by Harvey, for he says, "I confesse I have met with but little encouragement in this Designe, for all the Physicians and Anatomists I have hinted it unto have held it scarce feasible, Doctor Wright, Junior, only excepted . . . whose apprehension I found so well possessed with the gallantry (as he was pleased to speak) and the possibility thereof, that he promised me (to testify his approbation) he would commend it in his first public lecture of Anatomy in the College: a day much expected by those who had took notice of the most eminent and divine impulsions of his anatomic genius. But prevented by his much lamented death." If Robert Wright, Fludd's *protégé*, had lived to give his Goulstonian lecture in 1647 Bulwer's work might have been earlier recognised, and the expression of the Emotions might not have had to wait for their elucidation until the advent of Darwin. Where so much is good it is difficult to select an example of Bulwer's style, but the dissertation on kissing, if not the best, may still serve. It runs: "In Salutation, Valediction, Reconciliation or renewing of Love, Congratulation, Approbation, Adulation, Subjection, Confederation, but more especially and naturally in token of Love, we use to kiss which is done by drawing the lips into themselves and a little putting forth the parts that lie scattered loosely about the mouth, this being the usual prologue to a Kisse, which cannot be decently done unless we a little contract our mouth: which significations of our will are thus exhibited by the moving of the muscle, commonly called the Constringent paire of the lips, or Corrugans from puckering the mouth: which is done after this manner, the upper lip is not only drawn together but withal pulled downward, and the lower lip lifted up, whereby the lips are collected or reduced into themselves. This muscle I find from this employment to be called the Osculatorium, because it contracts the lips when we fasten a kisse upon another: which name implies only the manner of the outward action and not any inward affection of the mind exhibited thereby, the Latins having no word to signify both, which the Greeks have, with whom φιλεῖν is both to love and to kiss. This muscle from its office might be called the loving paire, Par Dilectionis, or the Sphincter of Salutation. Note that this muscle is assisted in this signification by the muscle Buccinator, which is common to the Lips with the Cheek, which muscle we have named the muscle of the Navell of Venus." The ideas contained in the book are clearly original, and Bulwer thinks that he "may with modesty suppose that I have sprung a new veine," and that he was the first "that by Art endeavoured to linke the muscles and the affections together in a new Pathomyogamia, or at least to have published the Banns between Myologus and Pathology."

